# Targeting under-screened women in cervical cancer: combining self-sampling and human papillomavirus testing with a strategic reminder plan

**DOI:** 10.1093/eurpub/ckaf122

**Published:** 2025-07-28

**Authors:** Sara da Graça Pereira, Luís Nobre, Marina Ribeiro, Patrícia Carvalho, Ana Morais, Rita Sousa, Ana Paula Moniz, Francisco Matos, Graça Fernandes, João Pedro Pimentel, José Carlos Marinho, José Luís Sá, Olga Ilhéu, Teresa Rebelo, José Fonseca-Moutinho, Hugo Prazeres, Rui Jorge Nobre, Fernanda Loureiro

**Affiliations:** Infogene, IPN Aceleradora, Coimbra, Portugal; Infogene, IPN Aceleradora, Coimbra, Portugal; Infogene, IPN Aceleradora, Coimbra, Portugal; Department of Public Health, Central Regional Health Administration (ARS Centro), Coimbra, Portugal; Working Group on Cervical Cancer in the Central Region of Portugal (GTCCU), Central Regional Health Administration (ARS Centro), Coimbra, Portugal; Salinas Family Health Unit, Praça Manuel Damião, Cacia, Portugal; Working Group on Cervical Cancer in the Central Region of Portugal (GTCCU), Central Regional Health Administration (ARS Centro), Coimbra, Portugal; Department of Gynaecology, Portuguese Institute of Oncology of Coimbra, Coimbra, Portugal; Working Group on Cervical Cancer in the Central Region of Portugal (GTCCU), Central Regional Health Administration (ARS Centro), Coimbra, Portugal; Department of Histopathology, Portuguese Institute of Oncology of Coimbra, Coimbra, Portugal; Department of Public Health, Central Regional Health Administration (ARS Centro), Coimbra, Portugal; Working Group on Cervical Cancer in the Central Region of Portugal (GTCCU), Central Regional Health Administration (ARS Centro), Coimbra, Portugal; Department of Histopathology, Coimbra Hospital and University Centre (CHUC), Coimbra, Portugal; Department of Public Health, Central Regional Health Administration (ARS Centro), Coimbra, Portugal; Working Group on Cervical Cancer in the Central Region of Portugal (GTCCU), Central Regional Health Administration (ARS Centro), Coimbra, Portugal; Working Group on Cervical Cancer in the Central Region of Portugal (GTCCU), Central Regional Health Administration (ARS Centro), Coimbra, Portugal; Santa Joana Family Health Unit, Aveiro, Portugal; Working Group on Cervical Cancer in the Central Region of Portugal (GTCCU), Central Regional Health Administration (ARS Centro), Coimbra, Portugal; Department of Gynaecology, Portuguese Institute of Oncology of Coimbra, Coimbra, Portugal; Working Group on Cervical Cancer in the Central Region of Portugal (GTCCU), Central Regional Health Administration (ARS Centro), Coimbra, Portugal; Department of Histopathology, Portuguese Institute of Oncology of Coimbra, Coimbra, Portugal; Working Group on Cervical Cancer in the Central Region of Portugal (GTCCU), Central Regional Health Administration (ARS Centro), Coimbra, Portugal; Department of Gynaecology, Coimbra Hospital and University Centre (CHUC), Coimbra, Portugal; Working Group on Cervical Cancer in the Central Region of Portugal (GTCCU), Central Regional Health Administration (ARS Centro), Coimbra, Portugal; Health Sciences Research Centre (CICS-UBI), Beira Interior University, Covilhã, Portugal; Infogene, IPN Aceleradora, Coimbra, Portugal; Center for Neuroscience and Cell Biology (CNC), Centre for Innovative Biomedicine and Biotechnology (CIBB), University of Coimbra, Coimbra, Portugal; Institute for Interdisciplinary Research (III), University of Coimbra, Coimbra, Portugal; Department of Public Health, Central Regional Health Administration (ARS Centro), Coimbra, Portugal; Working Group on Cervical Cancer in the Central Region of Portugal (GTCCU), Central Regional Health Administration (ARS Centro), Coimbra, Portugal

## Abstract

Cervical cancer (CC) screening is essential for reducing its incidence, yet engaging under-screened women remains challenging. Self-sampling has emerged as a promising solution to enhance attendance; however, its integration into programmes has proven difficult. This study evaluated a multimodal approach combining self-sampling, human papillomavirus (HPV) testing, and personalized contact to reach women not attending conventional CC screening. To achieve this, 801 women aged 30–59 who had not participated in Portugal’s Central Region CC screening programme for more than 4 years were selected based on specific criteria. Of these, 114 women were excluded for not meeting eligibility criteria, resulting in 687 eligible participants. Using an ‘opt-in’ approach, women who consented to participate received cervicovaginal self-sampling kits at home. Multiple contact strategies, including phone calls and reminder letters, were employed to encourage participation. Women testing positive for high-risk HPV (hr-HPV) were referred for gynaecological follow-up. Of the eligible women, 307 (44.7%) consented to participate and 198 (28.8%) provided valid samples for hr-HPV testing. Approximately 60.0% of participants were enrolled after the first reminder phone call, while additional contact strategies accounted for one-third of submitted samples. Among 12 hr-HPV positive cases, 11 completed gynaecological follow-up, resulting in the identification of six cervical lesions. This study confirms the feasibility and effectiveness of combining self-sampling, HPV testing, and personalized contact strategies to improve CC screening uptake among under-screened women. The findings highlight the potential of such interventions to address participation gaps and enhance early detection of cervical lesions, ultimately reducing CC incidence.

## Introduction

Cervical cancer (CC) remains a significant global health problem despite established screening programmes, vaccination, and treatment options. As the fourth most common and fatal cancer among women worldwide, CC claims the lives of over 340 000 women annually [1]. Portugal has been reported as the fourth-highest age-standardized incidence rate in Southern Europe in 2020, with 10.7 cases and 3.2 deaths per 100 000 women [2].

Organized CC screening programmes using cervical cytology have reduced global incidence and mortality rates [3]. However, limitations such as the low sensitivity and reproducibility of cytology [4], shortage of general practitioners, logistical difficulties, and some psychological barriers impede its effective implementation [5, 6]. In fact, most new CC cases are identified in women with infrequent or no screening history, often leading to later-stage diagnoses and more invasive treatments [7, 8].

The aetiological role of human papillomavirus (HPV) in CC is well established [9], and robust evidence supports the superiority of HPV testing over cytology in detecting high-grade cervical lesions, making it more effective in reducing disease incidence and mortality [10–13]. This method is highly reproducible and reliable and allows for longer screening intervals, especially for women who test negative for high-risk HPV (hr-HPV). This not only eases the burden on healthcare systems but also enhances overall screening efficiency [14]. Recognizing this advantage, the World Health Organization (WHO) now recommends the HPV test as the primary strategy for CC screening [15].

Despite efforts to implement organized CC screening programmes regionally, coverage rates in Portugal fell below expectations, with geographical coverage at 98.4% but annual population coverage at only 52.8% in 2019 [16].

Barriers to participation among Portuguese women include healthcare access issues, scheduling conflicts, lack of awareness, and sociocultural factors, with lower adherence observed in socioeconomically disadvantaged populations [17, 18]. Among immigrant women, attendance is further impacted by younger age, origin from Africa or Asia, marital status, and limited engagement with healthcare services [19].

The adoption of the HPV test as the primary method for CC screening has facilitated the introduction of self-sampling tests, allowing women to collect their own samples. This method overcomes critical barriers to CC screening, providing a convenient, private, and emotionally comfortable solution, potentially increasing screening attendance and reaching women who would otherwise not undergo screening [20]. Recent worldwide research consistently demonstrates that offering self-sampling kits for hr-HPV testing significantly increases screening numbers, particularly among women not reached by in-clinic programmes [21–24]. A meta-analysis involving a total of 482 271 women, concluded that self-sampling nearly doubled screening participation rates compared to clinician-collected samples (Relative risk: 1.8; 95% CI: 1.7–2.0) [25]. Multiple studies also confirm that self-sampling, especially when combined with hr-HPV PCR testing yields sensitivity for CIN2+ detection comparable to clinician-collected samples, thereby validating its efficacy in CC screening [22, 26, 27].

The present study represents a pioneering effort to develop strategies aimed at encouraging women who are not regular participants in the organized CC screening programme within Portugal’s Central Region. It combined the provision of self-sampling kits for hr-HPV detection with a strategic reminder plan to guide participants. The study employed an ‘opt-in’ approach, where only women who consented to participate were sent cervicovaginal self-sampling kits to their homes. Various strategies, including phone calls and follow-up letters, were implemented to remind women to respond to the invitation or submit their collected samples, with the goal of increasing participation rates. To guide this investigation, we formulated two key research questions:

1. Can a multimodal approach combining self-sampling for hr-HPV testing with personalized contact strategies effectively increase participation in CC screening among under-screened women?
2. Is this approach feasible and acceptable within the context of Portugal’s organized screening programme?

By addressing these questions, this study aims to provide relevant evidence on the potential value of integrating self-sampling with personalized outreach efforts to enhance the reach, equity, and overall effectiveness of national CC screening programmes.

## Methods

### Study design and sample selection

The present study received approval from the Ethics Committee of the Central Regional Health Administration (Study n° 23/2018) and was conducted from October 2018 to September 2019, predating the COVID-19 pandemic in the central region of Portugal.

A total of 801 women were randomly selected from the CC screening database of the Central Region of Portugal. To be included in the study, women had to meet the following eligibility criteria: (i) be alive at the time of contact and aged 30–59 years, (ii) have been unscreened for CC for at least 4 years, (iii) be non-pregnant at the time of contact, (iv) have an intact uterus and be medically able to perform self-sampling, and (v) reside in the country with valid address at the time of contact. Randomization was stratified based on six different Health Centre Groups (HCG): ‘Baixo Mondego’, ‘Baixo Vouga’, ‘Dão-Lafões’, ‘Pinhal Interior Norte’, ‘Pinhal Litoral’, and ‘Castelo Branco Local Health Unit’ (Table 1). Stratification ensured the representation of each individual HCG in the study population and three different age groups (30–39, 40–49, and 50–59 years).

**Table 1.** Study population characteristics: distribution of selected women by age group and HCGs

	Health Centre Groups						
Age (years)	Baixo Mondego	Baixo Vouga	Dão-Lafões	Pinhal Interior Norte	Pinhal Litoral	LHU Castelo Branco	Total
30–39	69	62	55	25	50	6	267
40–49	70	60	54	26	52	5	267
50–59	66	60	53	30	52	6	267
Total	205	182	162	81	154	17	801

Of note, Portugal’s screening guidelines currently recommend HPV testing every 5 years for women aged 25–60 [28]. However, at the date of present study (2018), cervical cytology triages every 3 years with HPV testing for atypical squamous cells of undetermined significance (ASC-US) cases was the protocol in force [29].

### Invitation letter, self-sampling kit, reminder phone calls A and B, and final reminder letter

**Figure 1.**
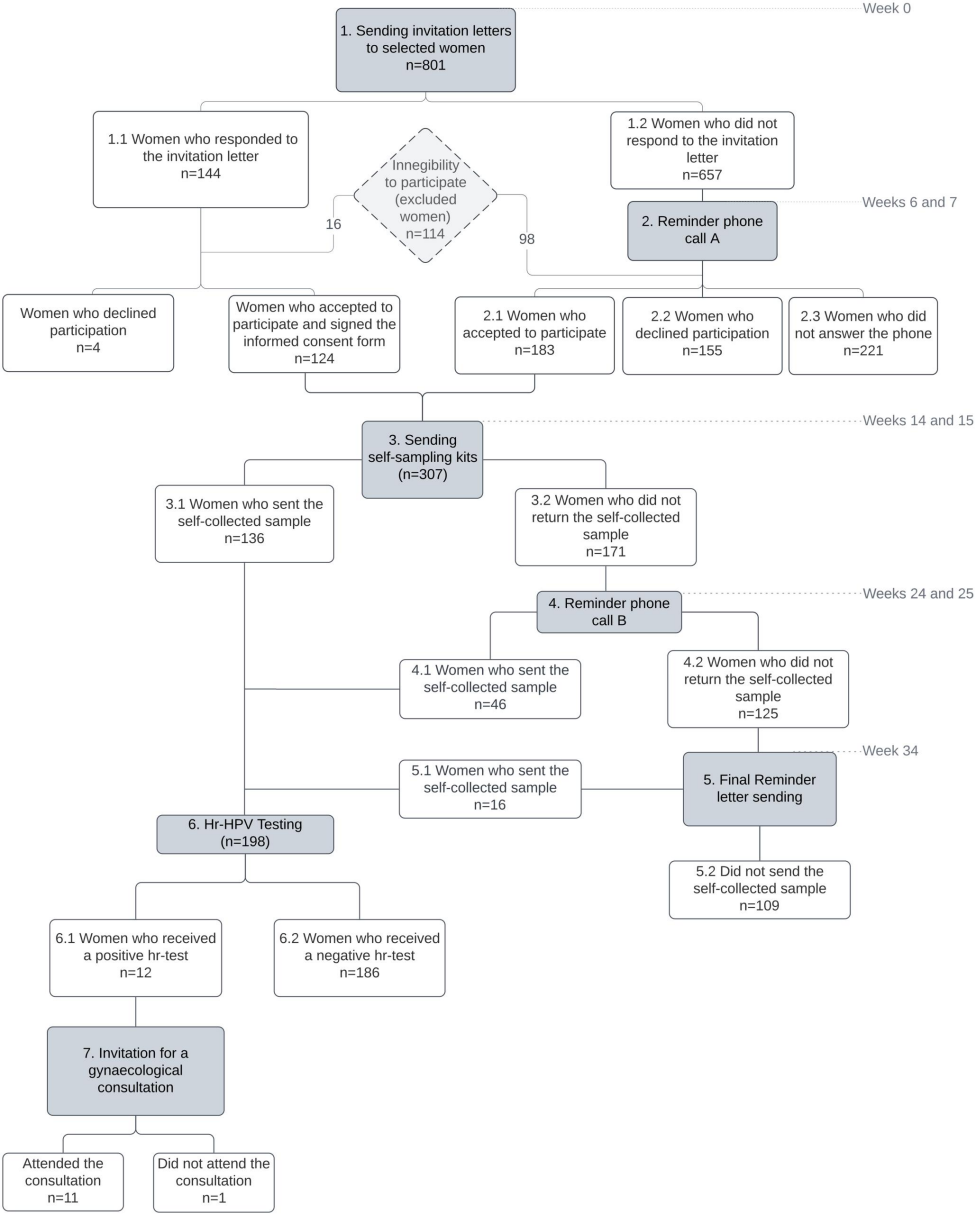
Study flowchart: involvement of participants throughout various phases of the study. This flowchart outlines the number of women who entered each phase of the study. To be included in the study, women had to meet the following eligibility criteria: (i) be alive at the time of contact and aged 30–59 years; (ii) have been unscreened for cervical cancer for at least 4 years; (iii) be non-pregnant at the time of contact; (iv) have an intact uterus and be medically able to perform self-sampling; (v) reside in the country with valid address at the time of contact.

The study flowchart is illustrated in Fig. 1. At the study’s onset (week 0), all selected women received an invitation letter outlining the study and an informed consent form. This allowed participants to confirm their interest in participating and request a self-sampling kit for cervicovaginal fluid collection, intended for subsequent hr-HPV testing.

Women who did not confirm their participation in 6 weeks after the invitation, a follow-up phone call (reminder phone call A) was conducted to confirm the receipt of the invitation letter (weeks 6 and 7 of the study). Women who expressed their interest in participating in the study, either by signing the informed consent form or during reminder phone call A, received a self-sampling kit during weeks 14 and 15 of the study. Each kit included a Qvintip sampler (*Aprovix AB, Solna, Sweden*), a labelled test tube with a cap, a data form, an instruction manual for self-sampling, storage, and shipment of the biological material, as well as a prepaid and pre-addressed envelope for sample shipment. Ten weeks after the kit distribution (weeks 24 and 25), women who had not yet sent the sample were contacted by phone for sample return—reminder phone call B. Finally, at week 34, a final reminder letter was dispatched to women who had not returned the biological sample, informing them of the study’s closure at week 41, after which no more samples would be accepted.

### Nucleic acid extraction and hr-HPV testing in self-collected samples

DNA was extracted from self-collected samples using the *RealLine DNA-Express kit—VBC8899-R* (Bioron Diagnostics GmbH, Römerberg, Germany). The presence of 12 hr-HPV types was detected by Real-Time PCR (qPCR) using the *RealLine HPV HCR Genotype Fla-Format—*VBD8482, a CE-IVD commercial kit assay (Bioron Diagnostics GmbH) following the manufacturer’s instructions, and the amplification was performed on the RealLine Cycler 96-5 thermocycler (Bioron Diagnostics GmbH). This test detects and identifies the following hr-HPV genotypes individually: 16, 18, 31, 33, 35, 39, 45, 51, 52, 56, 58 and 59. Additionally, it evaluates the quality of the extracted DNA by amplifying the endogenous beta-actin gene. A report with the hr-HPV test result (positive or negative) was sent to each of the women who submitted the self-collected sample.

### Gynaecology consultation, cytological diagnosis, hr-HPV testing, and anatomopathological examination

Women who tested positive for hr-HPV infection were referred to the colposcopy unit at the Portuguese Institute of Oncology in Coimbra 3 months after receiving their results. At their first visit, they repeated self-sampling using the Qvintip kit and underwent a gynaecological exam, including clinical evaluation, cervicovaginal sampling for cytology, and colposcopy with biopsy if any abnormalities were found. These procedures followed established best practices at the Gynaecological and Histopathology Laboratories of the Portuguese Institute of Oncology.

The detection and genotyping of hr-HPV infection from self-collected samples were conducted using the methodology outlined previously. Pap-test cytology and histology results were conducted by certified pathologists following the Bethesda classification system.

### Statistical analysis

Chi-square test and Chi-square test for trend were performed using Prism 9, version 9.5.1 (GraphPad Software, LLC). *P* values were reported for all tests to determine the statistical significance of the findings.

## Results

### Women’s participation and the impact of reminder strategies in the study

The main objective of the present study was to assess the feasibility of self-sampling combined with HPV testing as a complementary method to CC screening for women not regularly participating in it. A total of 801 Portuguese women from the central region, who had not undergone screening for 4 or more years, were invited to participate through an invitation letter.

Of the 801 women invited, 144 responded to the invitation letter, with the majority doing so within the first 2 weeks (Fig. 1 and Fig. S1). Among these, 124 women consented to participate in the study, 4 declined, and 16 were excluded due to not meeting the eligibility criteria. The remaining 657 women who did not respond within the first 5 weeks were subsequently contacted by telephone (reminder phone call A) during weeks 6 and 7 (Fig. 1 and Fig. S1). Among this group, 183 women agreed to participate, 155 declined, 221 did not answer the call, and 98 were excluded due to ineligibility.

In total, 114 women (14.2%) were excluded from the study due to not meet the eligibility criteria (Table S1). The majority of exclusions were due to recent participation in screening programmes (43/114, 37.7%), history of hysterectomy (32/114, 28.0%), or being absent from the country at the time of contact (19/114, 16.7%). The remaining exclusions, accounting for approximately 17.5% of ineligible cases, were due to pregnancy at the time of contact, underlying health conditions that precluded self-sampling, death, or invalid address or contact information (Table S1).

In response to the invitation letter and during reminder phone call A, reasons for non-participation were recorded for 159 women (Table S2). Of these, 36.5% reported already being monitored in the private sector, 3.1% cited lack of time, 3.8% expressed concerns about discomfort, pain, or doubts regarding correct self-sampling, 2.5% doubted the method’s effectiveness, and 2.5% preferred the traditional cytological examination.

Ultimately, 307 out of 687 eligible women (44.7%) agreed to participate in the study and requested the self-sampling kit (Fig. 1). It should be highlighted that more than half of these women (183/307, 59.6%) only decided to participate after receiving reminder phone call A, conducted during weeks 6 and 7 of the study (Fig. S1).

Of the 307 women who agreed to participate, 198 women (64.5%) collected and sent cervicovaginal fluid samples for subsequent hr-HPV testing. Of these, 136 samples were received within the initial 11 weeks after the distribution of self-sampling kits (in weeks 14 and 15 of the study), 46 samples were received following reminder phone call B at week 25 of the study, and an additional 16 samples were received upon sending the final reminder letter at week 34 of the study (Fig. S1). In summary, 31.3% of the samples received for HPV testing were collected after the implementation of reminder phone call B (46/198) and the final reminder letter (16/198).

Analysing the study’s population participation in each HCG, the highest participation rate was observed in the Dão-Lafões HCG (31.9%), followed by Baixo Mondego (30.7%), Baixo Vouga (28.3%), Pinhal Litoral (27.7%), and Pinhal Interior Norte (26.8%). No women from the ULS de Castelo Branco participated in the study (Table 2). However, statistical analysis using Chi-squared test revealed no significant differences regarding participation across HCGs (*P* values = 0.229). Regarding participation by age group, the data showed a slightly higher involvement rate among women in the ‘50–59 age group’ (32.4%), followed by the ‘40–49 age group’ (29.2%) and the ‘30–39 age group’ (25.1%) (Table 2). However, Chi-squared test showed no statistically significant association between participation and age group (*P* = 0.7849).

**Table 2.** Participation rate and hr-HPV prevalence across different health centres and age groups considered

	Total			Age group										
				30–39			40–49			50–59				
Health Centre Group	No of eligible women invited	Participants *n* (%)	Positive hr-HPV samples *n* (%)	No of eligible women invited	Participants *n* (%)	Positive hr-HPV samples *n* (%)	No of eligible women invited	Participants *n* (%)	Positive hr-HPV samples *n* (%)	No of eligible women invited	Participants *n* (%)	Positive hr-HPV samples *n* (%)		
Baixo Mondego	179	55 (30.7)	2 (3.63)	62	15 (24.2)	1 (6.7)	58	20 (34.5)	1 (5.0)	59	20 (33.9)	0 (0.0)		
Baixo Vouga	145	41 (28.3)	2 (4.9)	53	14 (26.4)	1 (7.1)	47	16 (34.0)	1 (6.3)	45	11 (24.4)	0 (0.0)		
Dão-Lafões	141	45 (31.9)	2 (4.44)	49	15 (30.6)	2 (13.3)	48	13 (27.1)	0 (0.0)	44	17 (38.6)	0 (0.0)		
Pinhal Interior Norte	71	19 (26.8)	1 (5.26)	22	6 (27.3)	1 (16.6)	22	6 (27.3)	0 (0.0)	27	7 (25.9)	0 (0.0)		
Pinhal Litoral	137	38 (27.7)	5 (13.1)	48	10 (20.8)	3 (30.0)	48	11 (22.9)	0 (0.0)	41	17 (41.5)	2 (11.8)		
Castelo Branco LHU	14	0 (0.0)	0 (0.0)	5	0 (0.0)	0 (0.0)	3	0 (0.0)	0 (0.0)	6	0 (0.0)	0 (0.0)	Comparison between ‘age’ groups	
All	687	198 (28.8)	12 (6.1)	239	60 (25.1)	8 (13.3)	226	66 (29.2)	2 (3.0)	222	72 (32.4)	2 (2.8)	Participation	hr-HPV
Comparison between HCG groups^a^	Chi-square/df	6.89, 5	4.258, 4		1.333, 4	3.503, 4		2.341, 4	1.766, 4		4.051, 4	6.655, 4	0.07447, 1	6.055, 1
	*P* value	0.229 (ns)	0.3722 (ns)		0.8558 (ns)	0.4775 (ns)		0.6733 (ns)	0.7787 (ns)		0.3991 (ns)	0.1553 (ns)	0.7849 (ns)	0.0139*

^a^ Comparisons were made using the Chi-square test; ns: non-significant.

* *P* < 0.05.

### HPV analysis results and cytological-histological evaluation of positive cases

Hr-HPV types were detected in 12 out of the 198 samples (6.1%). The incidence of hr-HPV infection was 13.3% for women in the 30–39 age group, 3.0% for women in the 40–49 age group, and 2.8% for women in the 50–59 age group, indicating that hr-HPV infection was less frequently detected in older women (Table 2). A Chi-squared test for trend revealed significant differences in the percentage of hr-HPV infections across age groups (*P* = 0.0139). In fact, women in the 30–39 age group had a significantly higher percentage of hr-HPV infections compared to the 40–49 and 50–59 age groups.

Women who tested positive for hr-HPV were referred to the colposcopy unit, with 11 out of 12 women attending (92.0% participation). The results of HPV tests from self-sampling procedures, cytology, colposcopic findings, and histological analysis are outlined in Table 3.

**Table 3.** Correspondence of hr-HPV test results in self-collected cervicovaginal fluid samples and in cytological samples and their correlation with cytological, colposcopic, and histological analyses

Age group	Woman ID	First hr-HPV test (self-sampling)	Second hr-HPV test (self-sampling)	Cytology	Colposcopy	Histology
30–39	64	HPV58	Negative	NILM	Normal	NP
	237	HPV45	HPV45	ASC-US	ACF (grade 1)	LSIL/CIN1
	397	HPV51	HPV51	ASC-US	ACF (grade 1)	LSIL/CIN1
	439	HPV16	HPV16	NILM	ACF (grade 2)	LSIL/CIN1
	571	HPV45	HPV45	ASC-US	ACF (grade 2)	Inflammatory alterations
	644	HPV31, HPV51	HPV31, HPV51	ASC-US	ACF (grade 1)	LSIL/CIN1
	674	HPV31, HPV33	HPV31, HPV33	ASC-H	ACF (grade 1)	HSIL/CIN2
40–49	138	HPV31	HPV31	NILM	Normal	NP
	308	HPV16	HPV16	ASC-US	ACF (grade 2)	LSIL/CIN1
50–59	749	HPV51	HPV51	ASC-US	Normal	NP
	754	HPV39	HPV39	NILM	Normal	NP

HPV: human papillomavirus; NILM: negative for intraepithelial lesion or malignancy; NP: not performed; ASC-US: atypical squamous cells of undetermined significance; ACF: abnormal colposcopic findings; ASC-H: atypical squamous cells, cannot exclude a high-grade squamous intraepithelial lesion; LSIL: low-grade squamous intraepithelial lesion; CIN1: cervical intraepithelial neoplasia grade 1; HSIL: high-grade squamous intraepithelial lesion; CIN2: cervical intraepithelial neoplasia grade 2.

Regarding the HPV tests conducted on self-sampling samples, there was nearly complete agreement (91.0%) between the results obtained from the first and second self-collected samples, except for one case (ID64), which turned negative. Cytological analysis identified six cases of ASC-US, one case of ASC-H, and four negative cases. Histological analysis revealed one case of non-neoplastic change (inflammatory alteration), five cases of CIN1 (mild dysplasia), and one case of CIN2 (moderate dysplasia).

In all cases with histological abnormalities, hr-HPV infection was detected in the corresponding self-collected sample.

## Discussion

Screening programmes play a pivotal role in reducing CC incidence and mortality globally [3]. Nevertheless, several countries, including Portugal, struggle with insufficient or non-existent CC screening coverage. In Portugal, only approximately 60.0% of eligible women undergo screening, leaving a significant percentage unscreened [16]. Since a significant proportion of CC cases occur in under-screened or never-screened women [8], it is important to encourage women to participate in screening programmes.

Various global studies have demonstrated that self-sampling significantly increases the involvement of women who are usually harder to reach in screening processes [21–24]. This method enables women to collect their own samples privately and conveniently, overcoming obstacles that typically prevent their engagement in CC screening. Meta-analyses and observational studies have also revealed that the sensitivity of HPV testing on self-collected samples, particularly when using PCR-based tests, is comparable to clinician-obtained cervical samples in detecting CIN2+ lesions [26, 27, 30, 31].

Our study marks the first attempt to test HPV self-sampling for CC screening among under-screened women in Portugal. The objective was to evaluate the impact of personalized contact strategies aimed at informing and reminding women to respond to screening invitation and, for those who agreed to participate, to submit their collected sample. The effectiveness of these strategies was assessed through improvements in participation rates and sample submission.

To achieve this, 801 women aged 30–59 who had not participated in the Central Portugal’s CC screening for more than 4 years were selected based on specific criteria. Among 687 eligible women, 307 (45%) accepted to participate in the study, and 198 (29%) collected and sent the cervicovaginal fluid sample. These rates surpass those reported in a recent systematic review and meta-analysis [14], which assessed various strategies including nine studies utilizing the ‘opt-in approach’, where participation rates ranged from 1.5% to 17.5%, with a pooled average of 8.5%.

The higher participation rate observed in our study may be attributed to the use of a personalized contact plan combined with self-sampling. Importantly, our findings highlight the critical role of engagement strategies in CC screening programmes. Approximately 60% of women decided to participate in the study following reminder phone call A, and about one-third of the collected samples were only submitted after reminder phone call B or the final reminder letter. This marked improvement in response rate highlights the efficacy of patient communication strategies as key points throughout the screening process. Indeed, other studies have also evaluated the importance of communication strategies in improving CC screening participation rates [32].

Despite the positive participation rates, it is important to note that our study employed an ‘opt-in’ approach, where the self-sampling kit was sent only to women who expressed interest, rather than distributing it to all eligible women. Studies have shown that the ‘send-to-all’ or ‘opt-out’ strategy, which involves delivering the self-sampling kits to all enrolled women, is more efficient in terms of participation rates [8, 14, 25, 26]. This strategy allows women to familiarize themselves with the self-sampling device before making their decision. Therefore, implementing an ‘opt-out’ strategy in the future could be beneficial in boosting participation rates and reaching a larger number of hard-to-reach women. Other successful strategies for increasing participation include community campaigns and door-to-door visits, which have demonstrated twice the participation rates compared to traditional invitation letters or reminders for standardized screenings [14, 26].

Overall, our data suggest that self-sampling is broadly acceptable across all age groups covered by the Portuguese screening programme. Although participation was slightly higher among women aged 50–59 years, followed by those aged 40–49 and 30–39, this trend was not statistically significant, suggesting that age did not substantially influence the likelihood of participation in this study. This finding aligns with a meta-analysis by Nishimura *et al.* [20], which evaluated age-related preferences across 25 studies. While some studies noted that younger women might prefer self-sampling due to reduced embarrassment, others suggested that older women might favour it due to negative experiences with traditional examinations. Overall, self-sampling is widely acceptable across diverse age groups.

Although a formal questionnaire was not utilized to explore reasons for non-participation, valuable insights were obtained during reminder phone call A and through responses to the invitation letter from women who declined to participate. Many reported that they were already engaged in routine monitoring within the private healthcare sector, which may reflect established care preferences and perceived continuity of care. Other common barriers included time constraints and concerns related to discomfort, pain, or uncertainty about performing the self-sampling procedure correctly. Additionally, some women expressed scepticism regarding the effectiveness of the self-sampling method or showed a preference for traditional cytological screening. These observations highlight the importance of understanding participant perceptions and logistical challenges, suggesting that future research incorporating comprehensive questionnaires could inform targeted strategies to enhance participation and overcome specific barriers.

Among the 198 samples received, DNA quality and hr-HPV infection were assessed. All samples were suitable for hr-HPV analysis, and 6.1% (12 cases) tested positive for hr-HPV. While lower than reported in other Portuguese studies [33, 34], this rate reflects our population, as we focused on women aged 30 and older, whereas previous studies included younger women. Still, our findings are consistent with a meta-analysis conducted by Arbyn and colleagues, which looked at 22 trials [26], and found hr-HPV prevalence rates among under-screened or never-screened women ranging from 6.0% to 29.4% in self-sampling strategies.

Among the hr-HPV-positive cases, women aged 30–39 exhibited the highest infection rate (13.3%), with significantly lower rates observed in the older age groups: 3.0% in the 40–49 cohort and 2.8% in the 50–59 age group. This pattern aligns with well-documented epidemiological trends showing a lower prevalence of hr-HPV infection in older women [2].

Out of 12 hr-HPV-positive women, 11 attended gynaecological follow-up, leading to the detection of abnormal colposcopic findings and histological confirmation of cervical lesions. This high follow-up rate highlights the potential of self-sampling to bridge the gap between initial screening and necessary medical intervention.

While our study offers valuable insights into the potential of self-sampling to improve CC screening rates in Portugal, certain limitations may affect the interpretation and broader applicability of the results. Our participant group was modest, starting with 801 women and narrowing to 687 eligible participants. This sample may not fully represent the diversity of women who do not participate or participate irregularly in screenings in Portugal.

The study also did not account for key sociodemographic factors such as race, religion, education, and economic status, which can significantly influence the success and acceptance of HPV self-sampling programmes [35–37]. Omitting these factors limits our ability to fully assess barriers and facilitators of self-sampling in Portugal. Moreover, the lack of a control group using conventional cervical cytology limits comparative analysis. Including such a group would have provided a clearer understanding of self-sampling’s effectiveness relative to traditional methods.

To enhance understanding, future studies should include a larger, more diverse participant pool and adopt a holistic approach that considers sociodemographic influences. This would help identify strategies to boost participation and improve screening outcomes.

The COVID-19 pandemic profoundly impacted healthcare, prompting lockdowns, resource reallocations, and postponement of non-urgent procedures, which disrupted preventive care across Europe. HPV vaccination and CC screening were particularly affected, raising concerns about long-term impacts on CC rates and mortality [38]. In response, the WHO endorsed primary HPV-based screening and included self-sampling in self-care guidelines [39], while the IARC updated recommendations to support self-sampling [40]. Amid these challenges, self-care strategies, including self-sampling for HPV testing, have emerged as essential solutions, addressing immediate needs while providing a sustainable approach to preventive healthcare.

In this context, our findings highlight the effectiveness of a multimodal approach that integrates self-sampling, HPV testing, and personalized contact strategies to enhance participation in CC screening programmes, particularly among under-screened women. While additional studies with larger and more diverse populations are needed, this approach shows promise as a complementary strategy to conventional screening methods, contributing to the reduction of CC incidence and mortality and supporting global efforts to improve healthcare outcomes.

## Supplementary Material

ckaf122_Supplementary_Data

## Data Availability

All data generated or analysed during this study are included in this published article and its supplementary information files. Key points A multimodal approach integrating self-sampling, HPV testing, and personalized contact strategies is effective in improving CC screening participation among under-screened women. Direct mailing of cervicovaginal self-sampling kits, combined with personalized follow-up strategies (phone calls and reminder letters), significantly increased sample submission rates. Approximately 30% of women who had not participated in CC screening for over 4 years submitted self-collected samples, with 12 hr-HPV positive cases identified. The study demonstrates the potential of this approach to detect cervical lesions early and contribute to the reduction of CC incidence in decentralized health regions.
