# Supplementary material for: Targeting under-screened women in cervical cancer: combining self-sampling and human papillomavirus testing with a strategic reminder plan
Source: Eur J Public Health. 2025 Jul 28;35(5):984–91. doi: 10.1093/eurpub/ckaf122 (PMC12529258; doi:10.1093/eurpub/ckaf122)
Supplement: ckaf122_Supplementary_Data [file ckaf122_supplementary_data.docx]

**Supplementary figure and table**

**
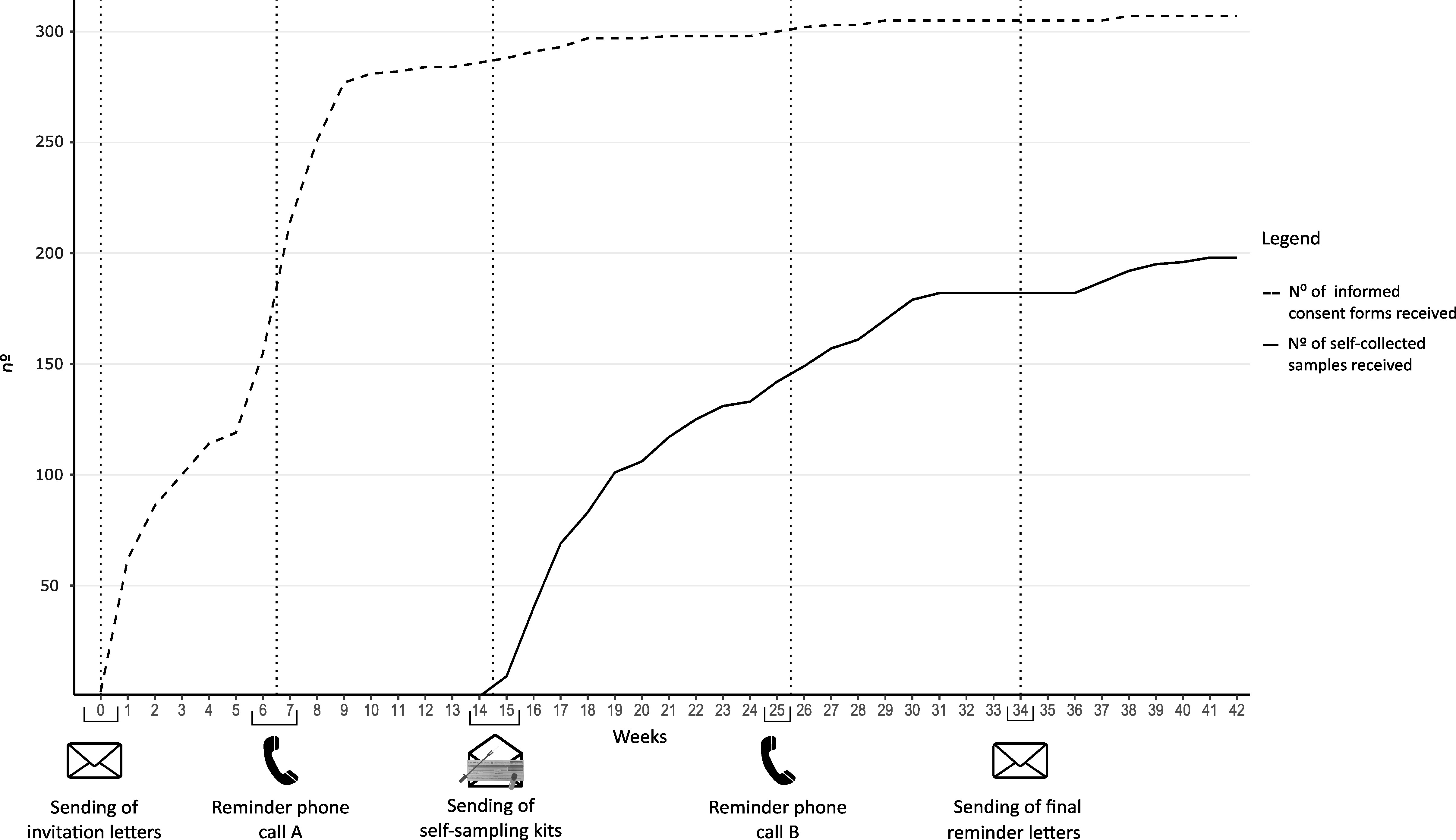
**

**Figure S1:** Correlation Between the Receipt of Informed Consents Forms, indicative of Participation Acceptance, and Subsequent Return of Self-Collected Samples.

**Table S1 - Reasons for Women's Ineligibility to Participate in the Study: Responses to the study invitation letter and Reminder Phone Call A**

| **Reasons** | **n (%)** |
| --- | --- |
| Participation in cervical cancer screening within the last 4 years | 43(37.7) |
| \| History of hysterectomy \| \| --- \|  \|  \| \| --- \| | 32(28.0) |
| \| Residing outside the country \| \| --- \|  \|  \| \| --- \| | 19(16.7) |
| \| Invalid address or contact information \| \| --- \|  \|  \| \| --- \| | 8(7.0) |
| \| Pregnant at the time of contact \| \| --- \|  \|  \| \| --- \| | 5(4.4) |
| \| Medically unable to perform self-sampling \| \| --- \| | 5(4.4) |
| \| Deceased at the time of contact \| \| --- \|  \|  \| \| --- \| | 2(1.8) |
| **Total** | 114(100) |

**Table S2 - Reasons Given by Women for Not Participating in the Study: Responses to the study invitation letter and Reminder Phone Call A**

|  | | n (%) |
| --- | --- | --- |
| **Logistical Reasons** | | |
|  | Attended elsewhere (abroad or private clinic) / Already had a scheduled appointment | 58(36.5) |
|  | Lack of time / Too busy | 5(3.1) |
| **Emotional or Attitudinal Reasons** | | |
|  | Fear / Discomfort / Pain / Previous unpleasant experience / Difficulty performing self-collection | 6(3.8) |
|  | Lack of trust in self-collection methods | 4(2.5) |
|  | Preference for cytology | 4(2.5) |
| **Unjustified Reasons** | | 82(51.6) |
| **Total** | | 159(100) |
